# An iconic traditional apiculture of park fringe communities of Borena Sayint National Park, north eastern Ethiopia

**DOI:** 10.1186/s13002-015-0051-1

**Published:** 2015-09-07

**Authors:** Hussien Adal, Zemede Asfaw, Zerihun Woldu, Sebsebe Demissew, Patrick van Damme

**Affiliations:** Department of Biology, College of Natural Sciences, Wollo University, P.O. Box, 1145, Dessie, Ethiopia; Department of Plant Biology and Biodiversity Management, College of Natural Sciences, Addis Ababa University, P.O. Box, 3434, Addis Ababa, Ethiopia; Laboratory for Tropical and Subtropical Agriculture and Ethnobotany, Department of Plant Production, Faculty of Bio-Science Engineering, Ghent University, Coupure links 653, 9000 Ghent, Belgium; Faculty of Tropical AgriSciences, Czech University of Life Sciences Prague, Kamycka 129, Prague 6, Suchdol 165 21 Czech Republic

**Keywords:** Apiculture, Bee forage, Floral calendar, Honey season, Response relationships

## Abstract

**Background:**

Traditional apiculture has been practised in Ethiopia over a long historical period and still remains a benign means to extract direct benefits from natural ecosystems. While its contribution to economic development and watershed protection is increasingly recognized its cultural significance is however, seldom noticed. This study was conducted using an ethnobotanical study approach to document the honey bee flora and associated indigenous knowledge of local communities in Borena Sayint National Park (BSNP), north eastern Ethiopia.

**Methods:**

Data were collected from 170 informants through semi-structured interviews and guided field walks, focus group discussion with 37 informants and 14 key informants and analyzed using standard analytical tools including ranking, comparisons and multivariate analyses.

**Results:**

In total, 152 bee forage species in 133 genera and 74 families were documented. The Asteraceae and Rosaceae were represented with six species each over the other plant families. Percentage of mentions per species ranged between 76.9 and 13.5 % for the most salient bee forage species. *Dombeya torrida*, *Erica arborea*, and *Olinia rochetiana* captured high community consensus as measured by rank order of popularity and designated as local appellation names of honey. Cluster analysis of priority ranking data showed relationships between key informants with respect to preferences, but ordination analysis did not indicate environmental proximity as a determinant of their responses. Five honey harvesting seasons occur each corresponding to the floral calendar of a dominant bee forage species that stipulate relocation of hives to appropriate locations within the national park.

**Conclusion:**

The apicultural tradition is iconic with economic value and forming part of the local peoples’ cultural identity apt to be preserved as a bequest for posterity.

## Introduction

Apiculture (*Syn*. beekeeping) remains one of the environment friendly long-standing modes of resource extraction from natural ecosystems. It uses honey harvesting as a benign means to extract direct benefits from natural ecosystems as well as managed landscapes neither causing disturbance nor compromising biodiversity [1]. In some parts of the world apiculture forms part of the work of hunter-gatherers, while elsewhere it is practised by highly industrialised agriculturalists in the world’s richest nations [2, 3]. The contribution of beekeeping in terms of the economic value that accrue from it, the ecosystem benefits it offers [4] and its worth to sustainable development has been increasingly recognized, but its cultural significance is seldom noticed.

In Ethiopia, traditional apiculture has been practised over a long historical period. Available records show that the practice as well as export of honey and beeswax has been established since the time of King Ezena who ruled the country in the third century AD [5]. As yet, there are evidences from the Hieroglyphs of ancient Egypt dating as far back as 5th century A.D. [6]. Over the centuries, the practice had passed down the generations while the resource was extracted using a traditional mode of extraction. In most traditional societies, the practice is still proceeding at the same pace. Nevertheless, this useful traditional practice has developed into an iconic cultural tradition offering certain societies unique cultural identity over the economic importance that is worth preserving in the future by putting in place strategies that enable survival of the culture.

Honey is a reliable source of national income from export [6] and of local income for small marginal farmers, women and other vulnerable members of society living surrounding natural forests, woodlands and riverine areas as well as a source of nutritious food, medicines and raw materials for pharmaceutical and cosmetic industries [3]. The current annual honey production in Ethiopia is estimated at approximately 24,000 tonnes contributing to about 24 % of the total for Africa and 2 % of the world honey production. The estimate is based on 65 and 75 % occupational efficiency of 7.5 million traditional and 20 thousand improved beehives respectively [5]. Furthermore, the country stands among the five biggest wax exporters to the world market given an average of 270 tonnes was exported per year over the period 1984–94 generating more than Ethiopian birr (ETB) 2,000,000 per annum to the national economy [7].

The promising local and national income gained from beekeeping activity has encouraged the government of Ethiopia to enhance the activity through launching an apiculture development program dealing with improving the bee management systems. Attempts are being made to introduce modern hives which are known to yield higher than the traditional hives. An average of 5–6 kg of honey could be collected per year from a traditional bees hive whereas the yield from an improved beehive may increase to 15–40 kg. Farm Africa attempted to implement beekeeping technologies around Chilimo State Forest as incentive for conservation of existing natural forests by providing beekeeping accessories and training packages to farmers engaged in beekeeping activities living in or around forestlands [8].

The Ministry of Agriculture and Rural Development (MoARD) has formulated a honey and beeswax development and marketing plan for the country. The Improving Productivity and Marketing Success (IPMS) of Ethiopian Farmers Project consider beekeeping as one of the prime activities producing priority marketable commodities in a number of its Pilot Learning districts/weredas [1]. The apiculture programme encourages community participation in conservation and local livelihood development over subsistence agriculture that has only little pay offs to improve the living standards of communities and rather leads to depletion of natural resources [9, 10]. Also, the Agricultural Sector Support Project (ASSP) has identified priority issues to improve the management of watersheds through categorization of land-use patterns involving beekeeping projects [9]. Supporting beekeepers with improved beekeeping technologies and providing access to market corridors encourages local efforts of biodiversity conservation and watershed protection [11, 12]. The support provides incentive for local people engaged in beekeeping to increase efforts of protecting natural stands of trees and integrate bee forage species known to improve honey production into managed fields [13].

Notwithstanding, the research attention given to apiculture so far in Ethiopia cannot be considered as good as its potential to contribute to sustainable forestry, watershed management and economic development. Few previous researches include assessing honey production and marketing systems [14, 15] compilation of honey bee plants and description of honey flow seasons [16, 17] and seasonal fluctuations of common bee flora [18]. Five hundred honey bee plants of various growth habits have been illustrated for the honey bee flora of Ethiopia [19]. Also, there are attempts to investigate the role of apiculture in protected areas watershed management and local income improvement [10]. But studies focussing on the ethnobotanical aspects of apicultural traditions held by indigenous communities living in and around protected areas are just meagre. This gap in research has therefore, prompted the current research carried out in BSNP. The objective of the study is to inventory the common honey bee flora of the national park, document the associated apicultural tradition of the park fringe communities and analyse the relationships occurring between key informants’ responses in their judgment of plant species providing for preferred bee forage. The results of this research will contribute to mainstreaming of beekeeping in the study area, enhance the traditional management practice to outstanding ecotourism value, a reliable means to conserve biodiversity and preserve the cultural heritage as a bequest.

## Materials and methods

### Description of the study area

**Fig. 1:**
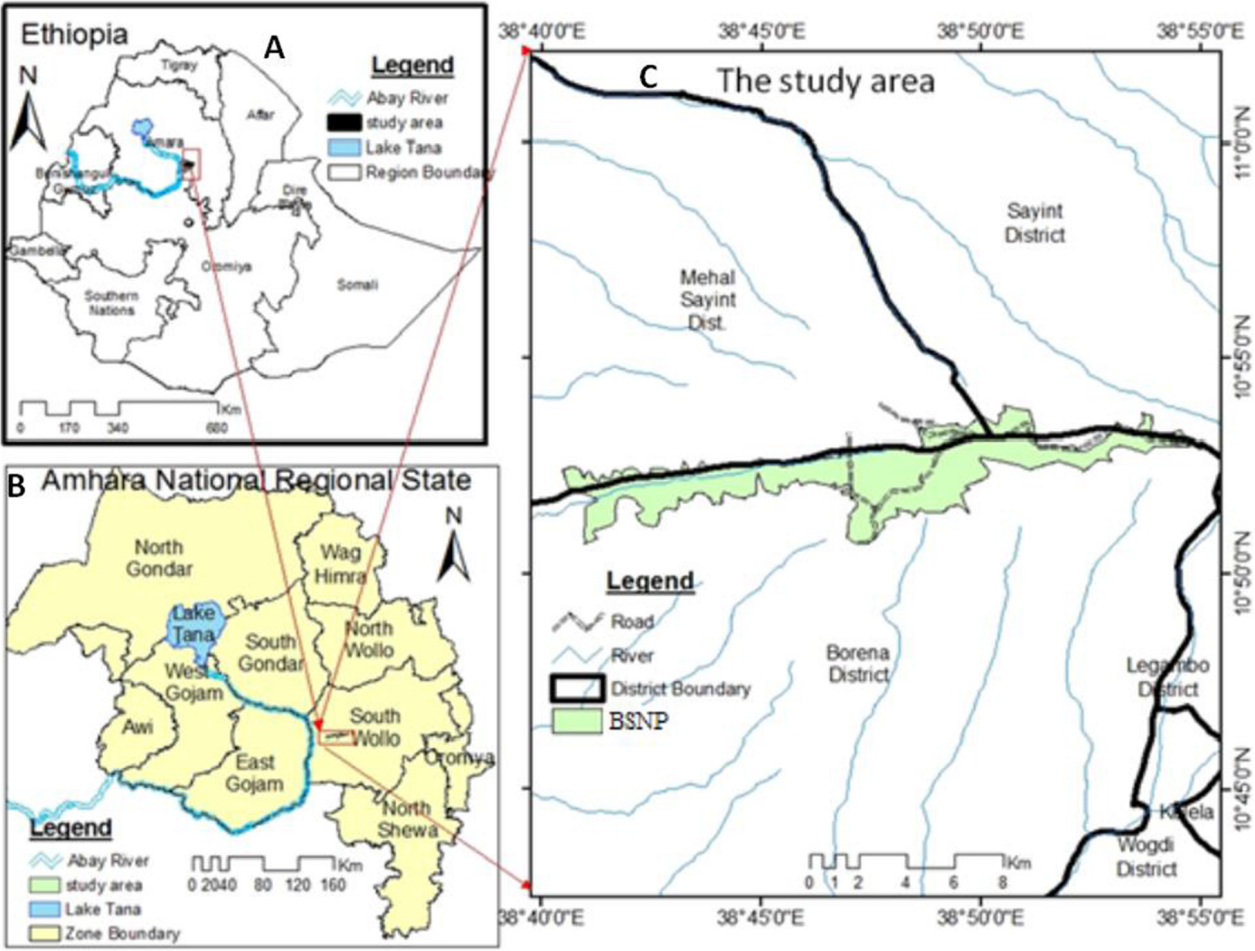
Map of the study area (**a**) Ethiopia, (**b**) Amhara National Regional State, (**c**) BSNP & adjoining districts [20]

The study area is located in the South Wollo Zone of the Amhara National Regional State in north eastern Ethiopia within the geographical coordinates of 10° 45′–11°N and 38°40′–38°55′E. It extends between 2188 and 3732 m altitudinal range forming part of the upper watershed of the Abbay River, the Ethiopian segment of the Blue Nile River. Two distinct vegetation zones occur below and above 3000 m altitudinal cut off point markedly responding to changes in altitudinal gradient. The Limesk Plateau sticks out above this cut off point separated from the adjacent low-lying settlement zone by sharp escarpments delimiting the subAfroalpine and Afroalpine vegetation hereafter referred to as GUASSA as named by the local people to indicate the dominance of Festuca spp. in the area. A dry Afromontane forest hereafter referred to as TIKUR DEN (commonly known as Denkoro Forest) is entrenched deep inside a large canyon sandwiched between two ridges of land masses partly forming the people-vegetation interface of Borena and Sayint weredas (districts). The BSNP is accessible through few entrance and exit gates opening to permanent footpath trails cross-cutting the vegetation in either direction. Before June 2009, its designation as a national park, the natural vegetation has long been subjected both to a heavy anthropogenic pressure and recurrent drought (Fig. 1) [20].

**Fig. 2:**
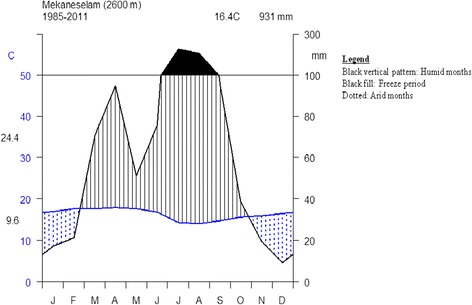
Climate diagram of Mekaneselam [21]

The rainfall regime of the study area is bimodal. A mean annual precipitation of 77.6 mm (sum of average monthly precipitation = 931.1 mm) and a mean annual temperature of 16 °C have been recorded over the years (1985–2011) (Fig. 2) [21].

Smallholder farming communities live off an agricultural mainstay on degraded slopes surrounding the national park. Outside peak farming seasons, the communities supplement their household income through selling their labour capital in nearby towns. The people inhabiting the Borena District interface trace their line of descent to a mixed Amhara-Oromo descent, Historical sources show that the Oromo group moved into the area from the Borena side during the end of the 16^th^ and the 1^st^ half of the 17^th^ centuries. The Sayint District had been inhabited by the Amhara group [22]. Sooner or later, the two ethnic groups got intermarried and integrated through various cultural exchanges, now making one linguistic and cultural group. The people depend on natural resources to satisfy their cultural and economic needs and have therefore, ever since influenced the natural vegetation of the study area.

### Sampling design

**Fig. 3:**
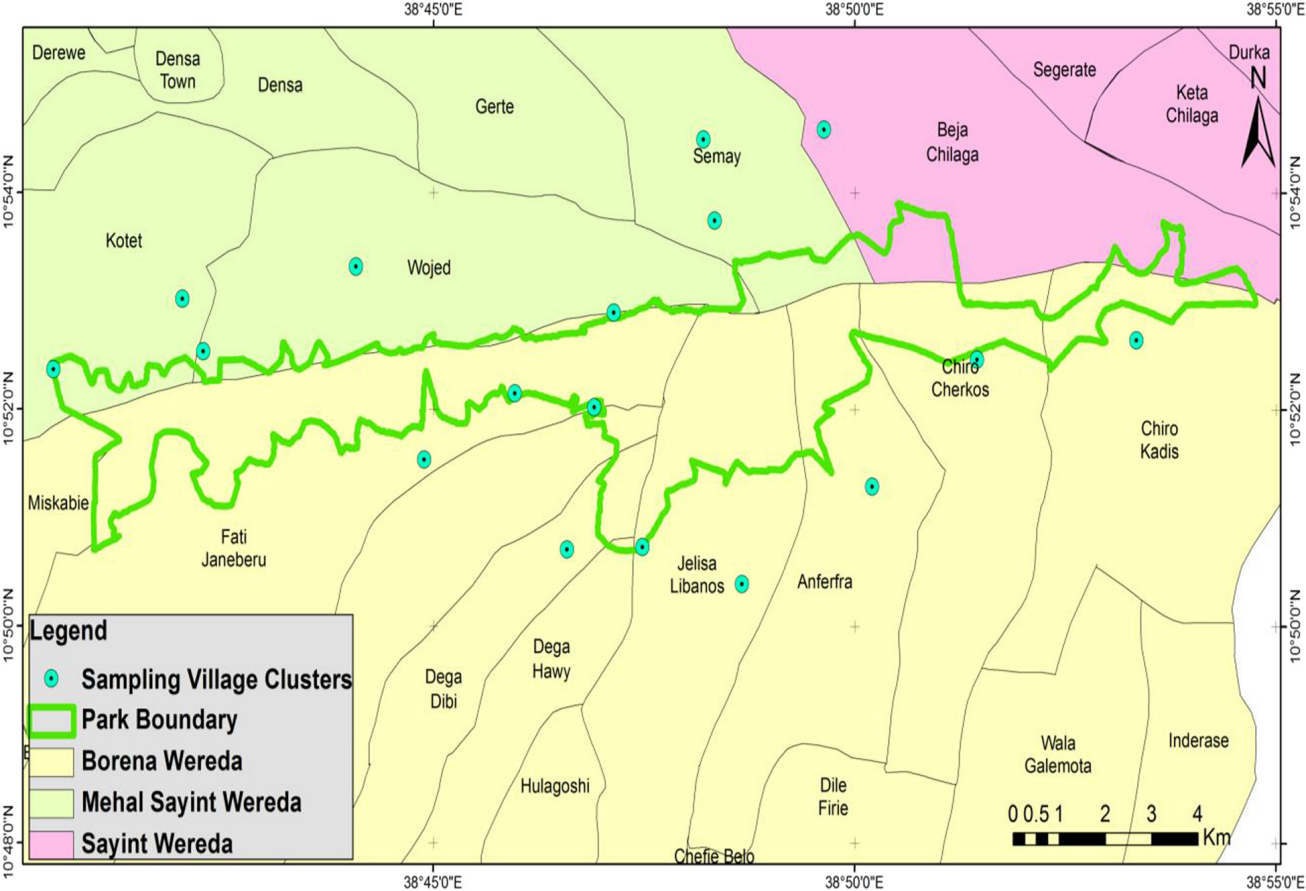
Map showing the ethnobotanical data sampling village clusters in the study area

The study population was estimated at 8290 individuals in 1190 households living in 35 park fringe village clusters of 13 Peasant Associations (PAs) found enclosing the national park interfaces on both districts. The total population of the 13 PAs surrounding the national park are estimated at 62,084 in 13,969 households [23]. Seventeen of the 35 village clusters were randomly selected by proportionate sampling to fairly distribute the sample size (Fig. 3). One hundred seventy informants, considered to be representative of the local community, were selected at 7 % precision level (e) and 93 % confidence interval. This was done by applying the Yamane’s formula [24] as modified by Cochran [25] *n* = N/(1 + Ne^2^) to the 1190 households making the 35 park fringe village clusters where: *n* = sample size, N = population, and e = sampling error (precision level) to get the overall local apicultural knowledge. Fourteen of 170 informants were selected based on their active role during the free listing exercises as evaluated by the researcher for the further undertaking of ranking and comparison exercises. Thirty seven informants (34 local inhabitants (2 × 17) plus 3 local elders), believed to have deep local environmental knowledge on the local apicultural tradition were selected by snowball sampling for focus group discussion. The verbal consent of each informant was obtained after explaining the purpose of the research. All plant voucher specimens were collected, identified and deposited at the Ethiopian National Herbarium (ETH).

### Data collection and analysis

A free list of the common bee forage plants was produced from informant citations using semi-structured interviews. Additional ethnobotanical data was gathered during focus group discussion, besides through intriguing questions with local elders and during guided field walks through forest transects and buffer zones. Pictures of relevant sites and objects were collected using an Olympus Master 2 camera. The most salient bee forage plants were determined based on rank order of popularity taking the values of each species’ saliency order. Further ranking and comparison exercises were carried out to generate quantitative data of which the result of the preference ranking exercise (preference ranking matrix) was subjected to multivariate analysis. Excel spreadsheet, EstimateS and R packages were used as data analytical tools. The former was used for the analysis of descriptive statistics while the latter two were used to draw species-informant curve, and depict cluster diagram (dendrogram) and ordination graph (scattergram) respectively.

## Results and discussion

### Diversity of bee forage species

**Fig. 4:**
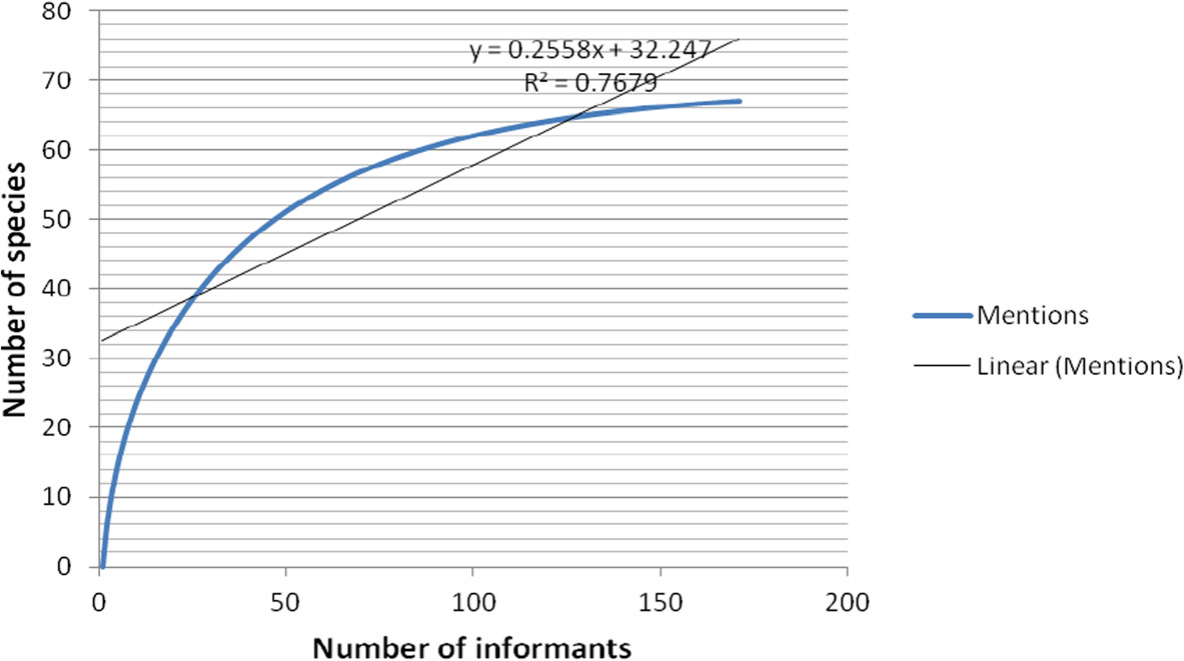
Species-informant curve for bee forage species in BSNP

Of 354 plant species reported in Adal [26], the people surrounding BSNP have cognitive domain for 152 bee forage species, all of them among the 500 important honey bee forage species illustrated in Fichtl and Adi [19]. Emerging practices of integration of apiculture with watershed protection projects [4, 9], the recognition of the contribution of crop plants pollination [2, 3, 10] and the location of BSNP at the upper watershed relative to the Great Renaissance Dam Project site implicates the significance of the bee forage species diversity in BSNP for initiating apiculture-related development projects. Sixty seven (19 %) species grouped in 60 genera and 41 families were locally perceived as more valuable bee forage plants (Appendix 1) than the remaining species. These are species immediately and more frequently listed during interviews and hence considered widely recognized by the community. Another group of 85 (24 %) species were recorded from intriguing questions posed to informants during guided field walks indicating that relatively more number of bee forage species was recorded through intriguing informants during guided field walks than it happened during free listing exercises. Some important bee forage plants might have been escaped from the lack of scrupulous free listing on the informants’ side due to the thinking that “honey bees visit almost any flowering plant”. However, as shown in Fig. 4, about 50 informants were sufficient to get the most popular (top 50) bee forage species of the area.

**Fig. 5:**
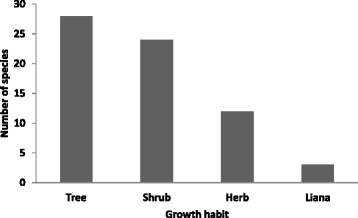
Distribution of bee forage species by growth habit

Members of the families Asteraceae and Rosaceae were represented by 6 species each; Acanthaceae 4 species; Fabaceae, Myrsinaceae and Oleaceae 3 species each and other families by 2 or 1 species. The most salient bee forage species were *Dombeya torrida*, *Erica arborea* and *Olinia rochetiana*. Judgement of the relative importance of bee forage species varied with agroecological context and the local peoples’ perception of the species with regards to bee forage value drawing from their acute observation of honey bees visiting the flower of the species. It has been reported that *Becium grandiflorum*, *Hypoestes forskaolii*, *Leucas abyssinica* and *Eucalyptus camaldulensis* are the major bee forage species in the eastern parts of Tigray [18] while *Hypoestes trifolia*, *Ocimum bacilicum*, *Becium grandiflorum*, *Guizotia abyssinica*, *Acacia seyal*, *Grewia bicolour* and *Eucalyptus camaldulensis*, in the Sekota District [17]. These are areas of lower altitudinal ranges than the current study area. Tree and shrub species account for higher percentages (42 and 36 % respectively) of the bee forage species in BSNP (Fig. 5) and this is expected because our research was undertaken in a forest ecosystem to a large extent. More tree and shrub species records in the current study contrasts with previous works [17, 27] which reported more herbaceous species implying the replacement of trees and shrubs by secondary forest herbs and cultivated crops.

### Saliency and local preference of bee forage species

Seventy seven to 13.5 % of mentions cited *Dombeya torrida*, *Erica arborea*, *Rosa abyssinica*, *Olinia rochetiana*, *Hagenia abyssinica*, *Apodytes dimidiata*, *Hypericum revolutum*, *Ekebergia capensis*, *Myrsine melanophloeos*, *Eucalyptus globulus*, *Vernonia rueppellii* and *Rubus steudneri* and so 9.4 to 0.6 % for the remaining species. The highest preferences were occupied by the 18 most salient bee forage species (Table 1).

**Table 1:** Matrix of preference ranking of 18 bee forage plants by 14 key informants (traditional apiculturalists)

Species	Key informants															
	R1	R2	R3	R4	R5	R6	R7	R8	R9	R10	R11	R12	R13	R14	Score	Rank
*Dombeya torrida*	18	17	17	18	18	18	17	17	17	17	18	18	18	18	246	1
*Erica arborea*	17	18	18	17	17	17	18	18	18	18	17	17	17	17	244	2
*Olinia rochetiana*	15	16	15	14	16	16	8	2	2	15	16	16	16	16	183	3
*Trifolium semipilosum*	14	14	2	16	14	13	16	15	16	14	15	12	13	7	181	4
*Vernonia rueppelli*	7	11	13	11	11	10	14	13	8	13	9	10	11	9	150	5
*Thymus schimperi*	13	3	8	13	10	8	12	16	12	10	12	7	10	12	146	6
*Lobelia rhynchopetalum*	16	13	7	6	13	11	13	8	11	11	14	8	7	4	142	7
*Ekebergia capensis*	11	9	16	8	15	15	6	6	4	8	3	13	6	15	135	8
*Hagenia abyssinica*	2	8	10	12	1	12	15	10	9	5	11	4	15	14	128	9
*Myrsine melanophloeos*	9	5	4	9	6	4	9	9	6	16	10	11	14	1	113	10
*Apodytes dimidiata*	1	15	14	3	12	14	3	4	5	3	4	14	5	13	110	11
*Buddleja polystachya*	10	6	3	4	5	9	10	14	15	12	8	2	2	8	108	12
*Hypericum revolutum*	6	10	6	5	2	7	7	12	7	6	13	6	8	10	105	13
*Nuxia congesta*	4	7	9	7	9	3	5	11	13	7	6	5	4	5	95	14
*Hypoestes forskaolii*	12	12	12	15	3	2	2	3	3	2	2	15	9	2	94	15
*Rubus steudneri*	8	4	5	0	8	6	4	7	10	9	5	3	12	6	87	16
*Myrica salicifolia*	5	1	1	1	7	5	11	5	14	4	7	1	3	3	68	17
*Scolopia theifolia*	1	2	11	2	4	1	1	1	1	1	1	9	1	11	47	18

Paired comparison conserved the rank order of *Dombeya torrida*, *Erica arborea*, *Olinia rochetiana* and *Myrsine melanophloeos*, but incongruence occurred for other bee forage species (Table 2). *Dombeya torrida*, *Erica arborea*, and *Olinia rochetiana* were frequently matched from triads presented to key informants in each triadic comparison run to select one of the most preferred bee forage species (Table 3). A step by step evaluation and triangulation of the most matched triadic pairs placed the species in their successive triadic rank order. This cross reference of key informants’ responses using different quantitative analytical tools identified the prominence of key informants’ preference for *Dombeya torrida*, *Erica arborea*, and *Olinia rochetiana* with regards to their local significance. Bee forage value is one criterion used to measure the economic importance of melliferous plants including tree species [28] and to identify local peoples’ preferences for trees in terms of their potential as good sources of nectar and pollen powder from which honey is made.

**Table 2:** Matrix of paired comparison of 10 of the most salient bee forage species

Species	Key informants															
	R1	R2	R3	R4	R5	R6	R7	R8	R9	R10	R11	R12	R13	R14	Score	Rank
*Dombeya torrida*	8	8	8	9	9	9	8	8	8	8	9	9	9	9	119	1
*Erica arborea*	9	9	9	8	8	8	9	9	9	9	8	8	8	8	119	1
*Olinia rochetiana*	6	7	4	7	7	5	6	0	2	7	7	7	7	7	79	3
*Ekebergia capensis*	7	5	6	6	6	7	4	2	2	5	1	6	1	5	63	4
*Vernonia rueppellii*	3	4	5	5	4	3	2	7	5	4	3	3	6	2	56	5
*Hagenia abyssinica*	2	3	1	3	0	3	4	6	5	3	4	1	5	6	46	6
*Myrsine melanophloeos*	5	1	2	1	3	1	4	3	3	5	5	4	5	1	43	7
*Hypericum revolutum*	1	3	3	2	2	2	1	6	6	2	6	2	3	3	42	8
*Apodytes dimidiata*	2	5	7	4	5	6	0	0	0	0	0	5	0	4	38	9
*Myrica salicifolia*	1	0	0	0	1	1	7	4	3	1	2	0	2	0	22	10

**Table 3:** Pooled matrix of 14 key informants’ triadic comparison of 6 most prefered bee forage species (Key: 1. *Dombeya torrida*, 2. *Erica arborea*, 3. *Olinia rochetiana*, 4. *Hagenia abyssinica*, 5. *Apodytes dimidiata*, 6. *Hypericum revolutum*)

Species	1	2	3	4	5	6	Rank
1							1
2	56						2
3	36	36					3
4	9	19	17				5
5	18	19	16	9			4
6	9	10	6	6	2		6

### Multivariate analyses of key informants’ response relationships

**Fig. 6:**
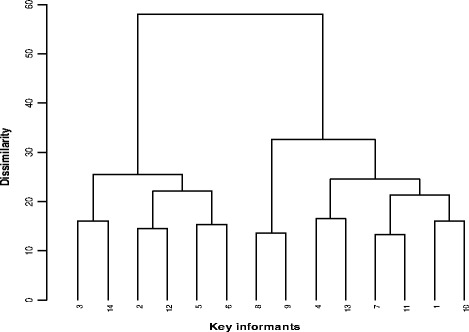
Dendrogram showing response relationships for preference of bee forage species

**Fig. 7:**
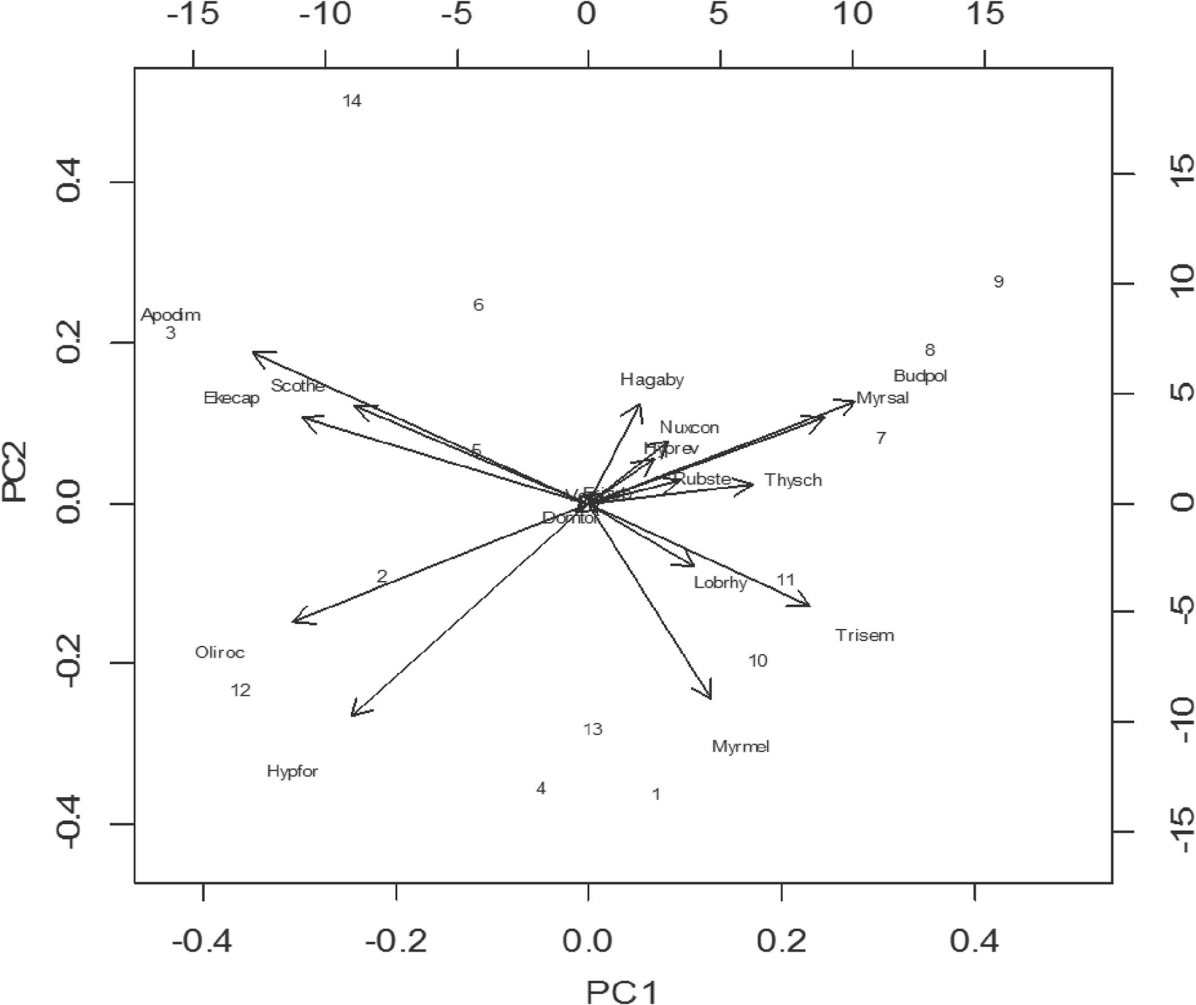
Biplot of 14 key informants in the preference ranking of 18 bee forage species (Key: (Apodim) *Apodytes dimidiata*, (Budpol) *Buddleja polystachya*, (Hagaby) *Hagenia abyssinica*, (Hypfor) *Hypoestes forskaolii*, (Hyprev) *Hypericum revolutum*, (Lobrhy) *Lobelia rhynchopetalum*, (Myrmel) *Myrsine melanophloeos*, (Myrsal) *Myrica salicifolia*, (Nuxcon) *Nuxia congesta*, (Oliroc) *Olinia rochetiana*, (Rubste) *Rubus steudneri*, (Scothe) *Scolopia theifolia*, (Thysch) *Thymus schimperi*, (Trisem) *Trifolium semipilosum*, (Verrue) *Vernonia rueppelli*)

Cluster and ordination analyses of the preference ranking matrix explained each key informant’s underlying attitude towards each bee forage species as it is also true of the inter-key informant response relationships in scoring preference ranks to 18 bee forage species presented to them for judgement. Lack of adequate related literature made it difficult to relate the observed configuration of the key informants’ in the dendrogram (Fig. 6) and scattergram (Fig. 7). In the dendrogram at linkage distance 25, two cluster solutions separate two groups [3, 14, 2, 12, 5, 6] and [8, 9, 4, 13, 7, 11, 1, 10] from influences of environmental proximity and a combination of other factors. Key informants [3, 14, 2, 12] live close to the margins of the TIKUR DEN vegetation while [8, 9, 4, 13, 7, 11, 1, 10] close to the GUASSA vegetation. But astonishingly, key informants 5 and 6 of the GUASSA group aligned differently in their responses with the TIKUR DEN vegetation group. Below Eucledian linkage distance 20, key informants are linked in a pair of two at varying distance levels; key informants 3 and 14, 5 and 6, 8 and 9 live in adjacent PAs of the same district-park interface, 2 and 12 live in adjacent PAs of different district-park interfaces. Thus, they ranked the local preference of the bee forage species in their localities more similarly. Some hidden variables must be responsible for the response similarity between other key informant pairs.

As observed from the separation of some of the key informants in the ordination graph from their cluster positions in the dendrogram (Fig. 7), the key informants’ grouping depicted in the cluster analysis is not consistent with the alignment of key informants in the ordination space. However, the projection (distance) of both key informants (objects) and descriptors (bee forage species) in the ordination axes have clued on the correlation of key informants’ preferences to particular bee forage species against their provenance and other variables. A maximum gradient length exceeding 4 standard deviation (sd) [29] and increasing distance from the origin (0, 0) in the direction of the gradient vector [30] implies a strong unimodal response between key informants in their judgment of the preference ranks of bee forage species as good source of nectar and/or pollen for honey bees. *Myrica salicifolia*, *Thymus schimperi*, *Trifolium semipilosum*, *Buddleja polystachya*, *Myrsine melanophloeos*, *Lobelia rhynchopetalum* and *Hagenia abyssinica* which are common in the GUASSA vegetation are projected farthest from the origin in their vector direction along PC1 closer to key informants of the GUASSA vegetation category implying that key informants of the GUASSA communities give similar judgement to these species. *Apodytes dimidiata*, *Scolopia theifolia*, *Ekebergia capensis*, *Olinia rochetiana* and *Hypoestes forskaolii* which are common in the TIKUR DEN vegetation are projected farthest from the origin along PC2 having received similar judgments from key informants of the TIKUR DEN vegetation. Thus, the observed projection can be taken as a proxy of more preference of key informants to the respective species. Conversely, either key informant groups have less preference to bee forage species projected at the rear end of the species vector direction with which they are aligned. However, two key informants from the GUASSA group (key informants 5 and 6) paradoxically aligned with key informants living close to the TIKUR DEN vegetation. This mixing up of key informants of both vegetation zones doesn’t warrant environmental proximity to overrule as a main factor in determining the response similarity/dissimilarity of key informants’ in their judgment of preferences to bee forage species. This is also reflected in the species hyperspace as corroborated by the ecological distribution of bee forage species (Appendix 2). Irrespective of their omnipresent distribution below and above the 3000 m altitudinal cut off point used to delineate the GUASSA and TIKUR DEN vegetation, *Buddleja polystachya*, *Myrica salicifolia*, *Myrsine melanophloeos* and *Thymus schimperi* are projected along PCA1 with key informants of the GUASSA vegetation than with the TIKUR DEN vegetation. This variation can be invoked for the lack of environmental proximity as a factor of response similarity between key informants. Thus, other variables such as differences in bee keeping experience might be responsible to influence the attitude of key informants towards particular bee forage species causing response dissimilarity between them. On the other hand, *Dombeya torrida*, *Erica arborea* and *Vernonia rueppelli* are projected near to or at short distances from the origin indicating little or no dissimilarity in response between informants of both the GUASSA and TIKUR DEN vegetation. These species received similar judgements from the key informants. The wide popularity drawing from the use of the vernacular names of the former two species as local appellation names of honey and the cross-cutting geographic distribution of the latter species could have influenced the observed response similarity among key informants in favour of these species.

### An iconic traditional apiculture

For the people living surrounding BSNP, traditional apiculture is a sideline engagement along crop production and livestock herding. As described in various sources [1, 2, 9] the importance of beekeeping for conservation and sustainable development has been long recognized in the study area. It has thus, become part of the cultural tradition of the local people in which interested farmers can be engaged for little or no cost. Capturing wild colonies of bees from an absconding swarm [17] is the labour capital that a farmer incurs for rearing honey bees. This is done by mounting an empty bee hive on a standing tree in the forest/alley/crop field/pasture or purchasing a colony of bees obtained in this way. Before it is mounted on a tree, the beehive must pass through common treatment procedures believed to help attract and drive the absconding swarm into it. This includes brushing the inner wall of the beehive with green stems of *Helichrysum conglobatum*/*Ocimum lamiifolium*, also smoking it with *Olea europaea*/*Otostegia integrifolia* and anointing the same with beeswax. Ejigu [14] reported a similar hive treatment practice from Enebse Sar Midir District (Amhara Region) and Amaro Special District in the Southern Nations, Nationalities and Peoples Region, Ethiopia.

**Fig. 8:**
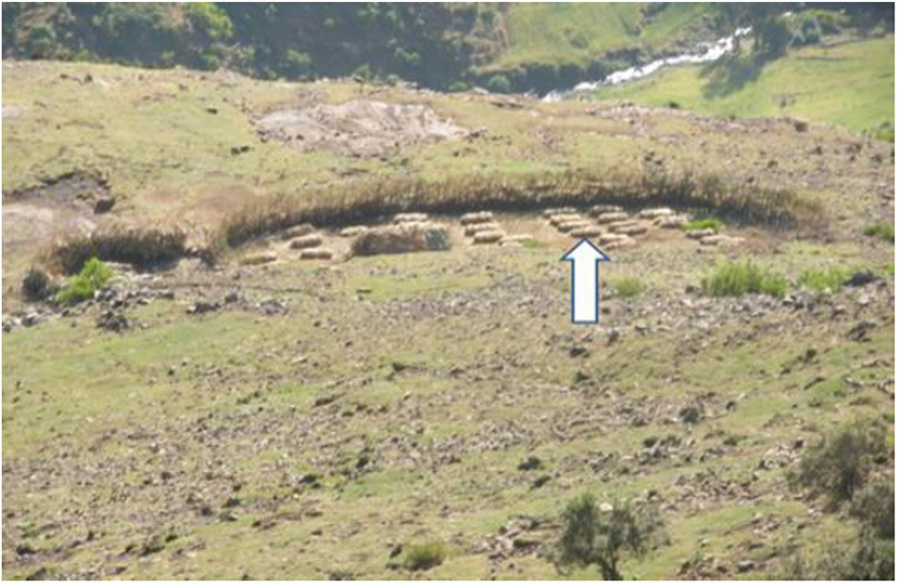
ZEDDA set at the National Park’s margin. *Arrow* points to a row of hives set up on site (Photo: Hussien Adal)

Two types of local honey bee farming systems occur both of which require confining the queen bee in a box that is placed inside the bee hive. In the sedentary bee farming system, few solitary traditional hives are often kept at the backyard or hung over a tree branch that is located away from home. In the mobile bee farming, a battery of hives, locally known as ZEDDA are set up at the forest margin in huge number owned ether by one individual or a group of individuals (Fig. 8). In the study area, ZEDDA is also used as a generic name to label places (toponyms) known to have been used as hive posts inside the forest in the past. The set up is fixed on a raised wooden lattice, its underneath dusted with ash to prevent insect infestation and fenced to prevent the set up from any interference. This important structure keeps the hive well above the ground and provides various functions. It helps to easily look out the hive, allow air circulation, prevent the action of strong wind and run-off, and avoid contact of the hive with insect pests. Once the traditional bee hives are placed in a good condition in this way, the remaining task of the beehive owners will be shadowing the ZEDDA.

The goal of setting ZEDDA is to bring honey bees closer to the forest treasure of nectar/pollen suffice to say that the local people have long realized the importance of keeping ZEDDA around apiary optima. The presence of 23 toponyms (Table 4) including 22 common toponyms and the historically important toponym known as NIGUS ZEDDA (King’s ZEDDA) indicates that each of these places have been named so after the owner of an abandoned ZEDDA. One of old ZEDDA located at Seddeta, a place found in the valley vegetation at the Sayint District interface bears the name of King Mikael of Wollo (1850–1918) that was known to have ruled Wollo and Tigre. The hive posts can continue as elements of ‘continuing cultural landscapes’ due to the fact that the evolutionary process of these cultural entities is still in progress retaining an active socio-economic role in the contemporary society as closely associated with their traditional way of life [31]. Moreover, selection of appropriate site with enough supply of bee forage within the flight range of honey bees [32, 33] and placing the hive within the carrying capacity of an area up to a radius of 3 km around the apiary for the bee to forage in one flight [34] is often recommended.

**Table 4:** Name of abandoned ZEDDA (old hive posts)

Name of ZEDDA	Meaning of name	Name of ZEDDA	Meaning of name
Nigus	King	Alemu Zeleke	After owner’s name
Mariam serka	St. Merry	Abi Legas	After owner’s name
Haro	Marshy place	Beyu Mitiku	After owner’s name
Kasa Gizaw	After owner’s name	Abebaw Adal	After owner’s name
Desalew Legas	After owner’s name	Tegegne Bire	After owner’s name
Yimer Abegaz	After owner’s name	Belete Fenta	After owner’s name
Yimer Muhie	After owner’s name	Tareke Tesema	After owner’s name
Wassie Gezahegn	After owner’s name	Tefera Kasa	After owner’s name
Sebsebe Abebe	After owner’s name	Abebaw	After owner’s name
Tesema Kibret	After owner’s name	Lulie Adal	After owner’s name
Adane Mengistu	After owner’s name	Arogew	Not currently used
Aragie Gizaw	After owner’s name		

### Floral calendar/local honey flow season

The strategy of the mobile beekeeping system developed in the study area involves shifting the position of the ZEDDA in response to a phenological signal elicited from the blossoming of a main bee forage species. This phenological signal serves to trigger bee keepers to set off their bee farming activities based on a floral calendar that struck at the flowering season of each main bee forage species. Identification of the flowering calendar of bee forage species helps to plan management practices like the time of honey harvest, seasonal shortfall of bee forage, measures of forage development [35] and protection of honeybee colonies against pests and diseases. Transferring beehives from place to place following the phenological succession of flowering plants is common in Amhara and Tigray Regions, northern Ethiopia [36, 14, 16, 17, 15].

In the current study area, there are five times of the year when the dominant bee forage species in the vegetation of the national park and the surrounding landscape blossom along which honey product can be harvested. This is two times over that reported in Jenberie [17] from Sekota District, northern Ethiopia as characterized by a major honey flow period occurring between August to October and a minor honey flow period between March to May. In parts of eastern Tigray [18, 14] Amaro District, southern Ethiopia and Enebse Sar Midir District, northern Ethiopia [14] beekeepers often move their hives twice a year to make an efficient use of the surrounding bee flora. This includes moving beehives from the highlands to the lowlands during the big rainy season (KIREMT) and when there is a shortfall of small rains (BELG). The presence of a succession of more number of floral calendar or blossoming season in BSNP compared to those areas with relatively few floral calendars so far recorded in Ethiopia makes the local traditional apiculture iconic of the local people. This uniqueness clearly distinguishes the people living around BSNP from other people engaged in beekeeping activities elsewhere in the country. Besides, it provides them with a sustainable means to efficiently utilize the bee forage species across temporal and spatial dimensions, engage local beekeepers in the mainstay over a longer period of the year and optimize economic benefits from the activity as well as maintain the biodiversity of the national park.

Using this locally developed multiple honeybee calendar as a blueprint, external organizations including the government, nongovernment or even private companies can introduce improved beekeeping technologies enhancing the local beekeeping practices through increased honey production and the creation of local market chains. Improved beekeeping technologies such as modern hives can be integrated with the built-in traditional apiculture system in which locally made beehives are used. The integration of locally made and modern beehives doesn’t compromise each other instead allows both to serve complementary purposes markedly contributing towards scaling up the local honey bee keeping activity. While use of modern beehives increases yield of honey harvest, maintenance of traditional beehives alongside the associated knowledge may have equal pay off from tourism besides preserving the heritage. Circulating beehives inside the national park based on the floral calendar was however, stopped at least temporarily along the establishment of the national park until it was just reopened for the local people very recently hearing the voice of the people and suggestions put forward in this research. The force measure was just against the aspirations of the national plan set to enhance beekeeping activity through the formulation of honey and beeswax development and marketing plans, as noted in Girma et al. [1].

The unique occurrence of five periods of floral calendar in BSNP, 3 occurring inside (Phase 1) and 2 outside (Phase 2) of the national park can be attributed to the temporal and spatial variations of honey bee species distribution in the natural vegetation, both in the national park and the adjoining geographic landscape. Phase 1 is set off at the end of August with setting up some ZEDDA in the Ericacious zone (GUASSA) following the blossoming of *Erica arborea* populations between September and October. The beehives are made to stay there until the end of October when honey is harvested. After the harvest of *Erica* honey, the beehives are transferred to a ZEDDA set up at the upper part of the Denkoro Forest where populations of *Dombeya torrida* dominate. Then, the beehives stay there until *Dombeya* honey is harvested between November and February. Next, between March and April, the beehives are transferred to the lower part of the Denkoro Forest responding to the blossoming of *Olinia rochetiana* populations. The harvest of *Olinia* honey at the end of April marks the end of Phase 1 for the year. Subsequently, the removal of the beehives from the forest at the end of Phase 1 switches the calendar over the second phase occurring outside the forest.

Phase 2 is set off when the flowers of *Olinia rochetiana* populations wither away roughly at the end of April. By then, the beehives are transferred to the adjacent WOINADEGA (warm to cool semi-humid) agroecological zone in response to the blossoming of *Eucalyptus globulus* populations where they stay until the end of June when Eucalypt honey will be harvested. The beehives are transferred *en route* to the lowlands or KOLLA (warm semi-arid climate) agroecological zones where *Bidens prestinaria* and *Guizotia scabra* form the main bee forage species at this time. At about the same time, resident beehives may be transported directly to the lowlands before July 5 often with herds of livestock which must be regularly moved to survive the hardy cold climatic condition of the DEGA (cold to cold humid) agroecological zone. The beehives stay at these destinations until brought back home around the 12^th^ of November. This is a deadline when beehives should be returned home due to the termination of the blossoming season of the major bee flora in the lowlands. The period overlaps with the blossoming season of *Dombeya torrida* populations in the upper Denkoro Forest (Phase 1). In the mean time, sedentary or residential beehives can make little honey from available resources.

**Fig. 9:**
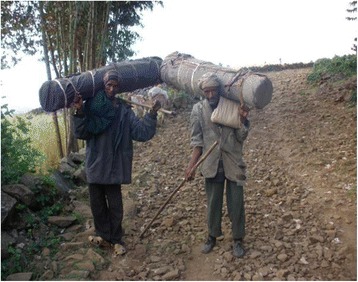
Two traditional apiculturalists transporting traditional beehives to a destination (Photo: Hussien Adal)

Shifting beehives inside the national park area when each phenological period sets off primarily requires selecting a convenient site at the edge of the forest where ZEDDA can be set up. Shifting beehives from the national park to the Eucalypt zone however, stipulates prior correspondence with someone willing to stay the hives in his backyard and observe them for the season. Lowland destinations are simply selected based on proximity to the national park fringe communities. As the work of transporting beehives gets started, the same containing honey bees are set out in the backyard and covered with some kind of cloth called SHEMMA then carefully carried away to the destination shoulder-high to avoid any possible breakage of honeycombs from mechanical shock (Fig. 9). The cloth helps to prevent rays of sun light penetrating the hive that cause bee leakage. Moreover, transportation of hives often occurs between dusk and dawn (6 pm and 6 am) when there can be no minimum doubt of sun rays entering the hive. Farmers at the Borena District interface move their beehives to the lowland lying along the Yeshum River watershed including Mirgaje, Workiemeskelie, Tikildingay, Sefatira, Dox, Agamsa, and Miskabe. At the Sayint District interface, areas located near the Abbay Gorge including Kotet, Dinecha, Asif, Woredeb, Derow and Zeqqa are the common sites where beehives are often moved to. Similar beehive relocation from the highlands to the lowlands occurring at the beginning of the main rainy season has been reported in the work of Ejigu [14]. At the destinations, the beehives are placed on site early morning after cleaning the site and filling the ground surface with ash.

### Local description of honey

The local experts make a quick analysis of honey using visual observation and simple physico-chemical parameters such as colour and texture to define the quality, characteristic and functional use of honey. In Sekota District, northern Ethiopia, local people have established three local categories of honey based on colour i.e. white, red and yellow [17]. The white honey is valued for table honey and income generation through market sale while the latter two are preferred for making mead. In BSNP, the local people associate honey stored in a container with a particular dominant bee forage species (Table 5) and also use the knowledge during market survey to distinguish between honeys stored in different containers. YE’ASTA MAR (*Erica arborea* honey), YE’WULKFA MAR (*Dombeya torrida* honey) or YE’TIFE MAR (*Olinia rochetiana* honey) are three common local names of honey denoting each of the three most salient bee forage species. Such an excellent local peoples’ beekeeping expertise and cultural tradition can be scaled up with supports of improved beekeeping technologies and provision of access to market corridors [11, 12] to engage the smallholders in the economic activity [3]. It can be used as an incentive to increase local efforts of protecting natural stands of trees and motivate the local people to grow selected tree species known to improve honey production [13] around buffer zones and settlement areas.

**Table 5:** Local description of honey

Species	Honey quality	Honey economics
*Dombeya torrida*, *Eucalyptus globulus*, *Apodytes dimidiata* and *Ekebergia capensis*	White	Table honey
*Olinia rochetiana*, *Erica arborea*, *Dombeya torrida* and *Eucalyptus globulus*	Crystallized	Table honey
*Erica arborea*	Crystallized like table sugar	Table honey
*Dombeya torrida*	Crystallized, white, sweetest	Table honey
*Guizotia scabra* and *Bidens prestinaria*	Brown	Mead known as TEJ/BIRZ
*Guizotia scabra*, *Bidens prestinaria*, *Euphorbia ampliphylla* and *Olinia rochetiana*	Sour	Mead known as TEJ/BIRZ

## Conclusion

Based on the results and discussion, the following conclusion has been made. Inventory of the BSNP flora recovered the common bee forage species. The local peoples’ cognitive domain for bee forage species is wide, but their cosmology discriminated only 67 (19 %) bee forage plants of the forest stock locally valuable in terms of their local significance as good bee forage resources. Of the total number of bee forage species recorded in the study, relatively more bee forage plants were listed from deeper interaction with informants as obtained during guided field walks than was so from the direct free listing exercises. The discrepancy could be accounted to the gap in informants’ overlooking of some of the plants during direct free listing exercises from the common viewpoint that “honey bees visit almost any flowering plant”. In terms of representation of taxa, members of the family Asteraceae and Rosaceae were highly represented in the bee forage species stock of BSNP. At species level, *Dombeya torrida*, *Erica arborea*, and *Olinia rochetiana* were the most salient, most preferred and used as local appellation names of honey as well. These species ruled out in all ranking and comparison exercises carried out to identify the most popular bee forage plants.

With regards to the observed response relationships between informants, the study revealed out that proximity to a particular vegetation category has a major influence on the informants’ response relationships. However, neither has the latter overruled as a main factor determining the overall similarity/dissimilarity of the key informants’ judgment in assigning priorities of preference to bee forage species nor has it influenced the alignment of bee forage species in relation to key informants. This is true because additional factors such as differences in bee keeping experience might be responsible to influence the attitude of key informants towards particular bee forage species thus, causing response variations between them. The traditional apiculture is iconic of the local people and much worth above its utilitarian value given its importance as a descriptor of the cultural identity of the local people. It is iconic in that it is guided by a local floral calendar set based on the local peoples’ collective knowledge of the phenological cycles of the main bee forage species growing in the area. However, emphasizing the implications of the local honey flow season that is based on the traditional apiculture is not to undermine the importance of introducing modern beekeeping technologies. Whereas keeping modern beehives increases yield of honey and accrues more income from market sale, parallel gains could be achieved from payments of tourists interested in traditional beehive management there by discounting the difference in the amount of total gains from beekeeping activities.

The local expertise expressed in testing the best quality honey during market survey and subsequent speculation of the raw material of its formation can be appreciated when it comes to its value as a tourism product. The study contributes to mainstreaming of beekeeping in the study area through bringing the value of the traditional apiculture to the immediate attention of local authorities, particularly the importance of maintaining and enhancing the traditional management practice. Moreover, the traditional apiculture contributes to the diversification of local livelihoods through income from market sale and tourism. This is besides guaranteeing the pollination of the crop plants growing in cultivated fields surrounding the national park. It is also a means to conserve biodiversity, protect the surrounding watersheds, and pass the traditional culture as bequest to the future generation. Hence, there is a need to conserve the national park flora and the associated iconic traditional apiculture.

## Appendix 1

**Table 6:** List of bee forage species recorded from free listing exercises (Key: *T* tree, *S* shrub, *C* climber, *H* herb, *FOS* found outside of site, *E* Endemic)

Vernacular name (Amharic)	Scientific name	Family name	Habit	Endemism	Col. no.
Nechilo	*Abutilon longicuspe* Hochst. ex A.Rich.	Malvaceae	S		320
Girar nech	*Acacia abyssinica* Hochst. ex Benth.	Fabaceae	T		15
Girar key	*Acacia negrii* Pic.-Serm.	Fabaceae	T	E	206
Shekori	*Acanthus sennii* Chiov.	Acanthaceae	S	E	4
Sesiy	*Albizia schimperiana* Oliv.	Fabaceae	T		30
Barimbs	*Allophyllus abyssinicus* (Hochst.) Radlk.	Sapindaceae	T		31
Eret wonde	*Aloe percrassa* Tod.	Aloaceae	H		300
Eret sete	*Aloe pulcherrima* Gilbert & Sebsebe	Aloaceae	H	E	313
Balamie	*Andropogon abyssinicus* Fresen.	Poaceae	H		116
Dong	*Apodytes dimidiata* E. Mey. ex Arn.	Icacinaceae	T		158
Azamir	*Bersama abyssinica* Fresen.	Melianthaceae	T		64
Adey abeba	*Bidens prestinaria* (Sch. Bip.) Cufod.	Asteraceae	H		11
Nech anfar	*Buddleja polystachya* Fresen.	Loganiaceae	S		107
Agam	*Carissa spinarum* L.	Apocynaceae	S		12
Kawot	*Celtis africana* Burm. f.	Ulmaceae	T		70
Azoareg	*Clematis simensis* Fresen.	Ranunculaceae	C		8
Lalinch	*Commelina benghalensis* L.	Commelinaceae	H		74
Bisana	*Croton macrostachyus* Del.	Euphorbiaceae	T		28
Chacha	*Cyanotis barbata* D.Don	Commelinaceae	H		342
Ameraro	*Discopodium penninervium* Hochst.	Solanaceae	S		60
Wulkfa	*Dombeya torrida* (J.F. Gmel.) P. Bamps	Sterculiaceae	T		103
Koshim	*Dovyalis abyssinica* (A. Rich.) Warb.	Flacourtiaceae	S		143
Gendero	*Echinops macrochaetus* Fresen.	Asteraceae	H		201
Sembo	*Ekebergia capensis* Sparrm.	Meliaceae	T		34
Asta	*Erica arborea* L.	Ericaceae	S		133
Bahirzaf	*Eucalyptus globulus* Labill.	Myrtaceae	T		161
Yekebir kulkual	*Euphorbia ampliphylla* Pax	Euphorbiaceae	T		270
Shola	*Ficus sur* Forssk	Moraceae	T		97
Warka	*Ficus sycomorus* L.	Moraceae	T		86
Wude	*Galiniera saxifraga* (Hochst.) Bridson	Rubiaceae	T		89
Lenquata wonde	*Grewia ferruginea* Hochst. ex A. Rich.	Tiliaceae	S		73
Koso	*Hagenia abyssinica* (Bruce) J.F. Gmel.	Rosaceae	T		153
Yeset milas	*Hygrophila schulli* (Hamilt.) M.R. & S.M. Almeida	Acanthaceae	H		84
Amja	*Hypericum quartinianum* A. Rich.	Hypericaceae	S		FOS 367
Amja	*Hypericum revolutum* Vahl	Hypericaceae	S		165
Tay matebia	*Hypoestes forskaolii* (Vahl) R. Br.	Acanthaceae	H		13
Kulsh, Woinagift	*Inula confertiflora* A. Rich	Asteraceae	S	E	195
Tero areg	*Jasminum abyssinicum* Hochst. ex DC.	Oleaceae	C		150,214,290
Alashume	*Laggera tomentosa* (Sch. Bip. ex A. Rich.) Oliv.& Heirn	Asteraceae	S	E	27
Jibera, Gemera	*Lobelia rhynchopetalum* Hemsl.	Lobelliaceae	H	E	267
Akelaho	*Maesa lanceolata* Forssk.	Myrsinaceae	S		20
Kombel	*Maytenus arbutifolia* (A. Rich.) Wilczek	Celastraceae	S		18
Atat	*Maytenus gracilipes* (Welw. ex Oliv.) Exell subsp. arguta (Loes.) Sebsebe	Celastraceae	S		23
Koladi	*Mimusops kummel* A. DC.	Sapotaceae	T		281
Shinet	*Myrica salicifolia* A.Rich.	Myricaceae	T		3
Gewra	*Myrsine melanophloeos* (L.) R. Br.	Myrsinaceae	T		209
Kechemo	*Myrsine africana* L.	Myrsinaceae	S		6
Asquar	*Nuxia congesta* R.Br. ex Fresen.	Loganiaceae	T		155
Woira	*Olea europaea* L. subsp. *cuspidata* (Wall. ex G.Don) Cif.	Oleaceae	T		82
Tife	*Olinia rochetiana* A. Juss	Oliniaceae	T		138
Tunjit geram	*Otostegia fruticosa* (Forssk.) Schweinf. ex Penzig	Lamiaceae	S		136
Tunjit eshoh	*Otostegia integrifolia* Benth.	Lamiaceae	S		FOS 373
Derg	*Phaulopsis imbricata* (Forssk.) Sweet.	Acanthaceae	H		47
Endod	*Phytolacca dodecandra* L’Herit.	Phytolaccaceae	S		170
Tikur inchet	*Prunus africana* (Hook. f.) Kalkm	Rosaceae	T		36
Ashkamo /Tatesa	*Rhus glutinosa* A.Rich subsp. *abyssinica* (Oliv.) M. Gilbert	Anacardaceae	T		17,277
Gurarba	*Rubus steudneri* Schweinf.	Rosaceae	S		147
Encholla	*Rubus volkensii* Engl.	Rosaceae	S		288
Embuacho	*Rumex nervosus* Vahl	Polygonaceae	S		10
Getem	*Schefflera abyssinica* (Hochst ex A. Rich.) Harms	Araliaceae	T		76
Wanaye	*Scolopia theifolia* Gilg	Flacourtiaceae	T		53
Worer	*Teclea nobilis* Del.	Rutaceae	T		37
Tosign	*Thymus schimperi* Ronniger	Lamiaceae	H		119
Chemekot	*Trifolium semipilosum* Fresen.	Fabaceae	H		126,213
Ketetina	*Verbascum sinaiticum* Benth.	Scrophulariaceae	H		295
Grawa	*Vernonia amygdalina* Del.	Asteraceae	T		333
Buyte	*Vernonia rueppellii* Sch. Bip. ex Walp.	Asteraceae	S		328

## Appendix 2

**Table 7:** Ecological distribution patterns of 18 bee forage species (+ present, – absent)

Scientific name/altitude (m)	2100	2200	2300	2400	2500	2600	2700	2800	2900	3000	3100	3200	3300	3400	3500	3600	3700
*Apodytes dimidiata*	–	–	+	+	+	+	+	+	–	–	–	–	–	–	–	–	–
*Buddleja polystachya*	+	+	+	–	–	–	–	–	–	+	+	+	–	–	–	–	–
*Dombeya torrida*	–	–	+	+	–	+	+	+	+	+	+	+	+	–	–	–	–
*Ekebergia capensis*	–	–	+	+	–	+	+	–	–	–	–	–	–	–	–	–	–
*Erica arborea*	–	–	–	–	–	–	+	+	+	+	+	+	+	+	+	+	+
*Hagenia abyssinica*	–	–	–	–	+	+	+	+	+	+	+	+	+	–	–	–	–
*Hypericum revolutum*	–	+	+	–	+	+	+	+	+	+	+	+	+	+	+	+	–
*Hypoestes forskaolii*	–	+	+	+	+	+	+	–	–	–	–	–	–	–	–	–	–
*Lobelia rhynchopetalum*	–	–	–	–	–	–	–	–	–	–	–	+	–	+	+	+	+
*Myrica salicifolia*	–	+	+	+	–	+	+	+	–	+	+	+	–	–	–	–	–
*Myrsine melanophloeos*	–	–	–	–	–	+	+	+	+	+	+	+	+	–	–	–	–
*Nuxia congesta*	–	–	+	+	+	+	+	+	+	+	–	–	–	–	–	–	–
*Olinia rochetiana*	–	+	+	+	+	+	+	+	–	–	–	–	–	–	–	–	–
*Rubus steudneri*	–	+	–	–	+	+	+	+	+	+	+	+	–	+	+	–	–
*Scolopia theifolia*	–	+	+	+	+	+	+	–	–	–	–	–	–	–	–	–	–
*Thymus schimperi*	–	–	–	–	–	–	+	+	+	+	+	+	+	+	+	–	–
*Trifolium semipilosum*	–	–	–	–	–	–	–	–	–	–	+	+	+	–	+	–	+
*Vernonia rueppelli*	+	–	+	+	+	+	–	+	–	+	+	+	–	–	–	–	–

## Abbreviations

AD: Anno domini
ARSD: Apiculture research strategy document
ASSP: Agricultural sector support project
BSNP: Borena sayint national park
ETB: Ethiopian Birr
ETH: Ethiopian National Herbarium
IPMS: Improving productivity and marketing Success
MoARD: Ministry of agriculture and rural development
PCA: Principal component analysis
PAs: Peasant associations
